# AI may enable robots to make a clinical impact in total knee arthroplasty, where navigation has not!

**DOI:** 10.1002/jeo2.70061

**Published:** 2024-10-19

**Authors:** Michael T. Hirschmann, Rüdiger von Eisenhart‐Rothe, Heiko Graichen, Stefano Zaffagnini

**Affiliations:** ^1^ Department of Orthopaedic Surgery and Traumatology Kantonsspital Baselland Bruderholz Switzerland; ^2^ Department of Clinical Research, Research Group Michael T. Hirschmann, Regenerative Medicine & Biomechanics University of Basel Basel Switzerland; ^3^ Department of Orthopaedics and Sport Orthopaedics University Hospital rechts der Isar, Technical University Munich (TUM) Munich Germany; ^4^ Department of Personalised Orthopaedics (PersO) at Privatklinik Siloah Berne Switzerland; ^5^ Department of Orthopaedic Surgery and Traumatology Clinica Ortopedica e Traumatologica II, IRCCS Istituto Ortopedico Rizzoli, c/o Lab Biomeccanica ed Innovazione Tecnologica Bologna Italy; ^6^ DIBINEM, University of Bologna Bologna Italy

**Abbreviations**
AI: artificial intelligence
TKA: total knee arthroplasty

In the last 10 years, total knee arthroplasty (TKA) has been and currently is undergoing a dramatic change in terms of enabling technology, which includes the introduction of robotic‐assisted surgery and the prospective incorporation of artificial intelligence (AI) in prediction modelling, planning and execution of robotic TKA [2, 7, 8, 9, 10, 12, 19, 20].

With the introduction of any enabling technology in the operating room (OR) such as navigational systems, a variety of clinical improvements and logistical benefits have been promised. In fact, navigated TKA did not hold all the promises and in fact a true clinical benefit for the patient by adding technological assistance, besides an improved accuracy and less outliers in comparison to conventional TKA, has hardly been demonstrated. Hence, after a great momentum for navigated TKA, it was abandoned by most of the European knee surgeons. In contrast, the Australian registry shows lower revision rates for patients <55 years and an increase in use of navigation systems. Now, next‐generation navigation systems, that is robotic systems, have taken over.

Nowadays, the use of robotic TKA again promises increased accuracy and improved patient outcomes. However, the main and pertinent question remaining is can robotic‐assisted TKA now hold the promise that navigation‐assisted TKA did not hold?

In fact, robotic TKA shows a higher accuracy than conventional TKA and navigation‐assisted TKA [1, 17]. The robotic arm, which is guided by preoperative imaging and planning, contributes to the process of ensuring that the implant is positioned in the precise location and alignment designed for it. Because of this precision, the chance of malalignment, which is a common cause of implant failure and required revision surgery, can be reduced. However, aside from the navigation, how much the robotic part really adds is still a matter of debate. An unanswered question still remains: How much does the more accurate execution of bone cuts add to the overall accuracy and outcomes of patients after TKA? In a more recent large database analysis, a reduced complication rate and reduced costs were found for robotic‐assisted TKA [11].

A more personalized approach to the individual knee is the most important cornerstone for improvement of patients' outcomes after robotic TKA [3, 4, 5, 6, 13, 14, 15, 16, 18]. The alignment and balancing strategy for the individual knee should be based on large database evidence connecting intraoperative decisions of bone cuts and balancing with postoperative outcome scores. For example, in a straight (neutral) knee with a 90–90° femoral–tibial joint‐line orientation the alignment strategies, whether mechanical, kinematic, restricted kinematic alignment (KA), inverse KA or functional alignment are not different for the coronal plane and show minor differences in the sagittal and rotational plane [14, 15]. Contrarily, in a varus or valgus phenotype, the differences dependent from the alignment philosophy chosen are more pronounced and clinically decisive [14, 15]. While current alignment strategies focus on coronal alignment, future 3D phenotype alignment strategies will include sagittal and rotational plane. This will increase the number of decision parameters enormously. Future, AI‐driven guidance will allow, not only to preplan the surgery in 3D but also to perform a simulation of the operation in advance and finally execute it.

As robotic systems continue to advance, it is anticipated that they will incorporate AI and machine‐learning algorithms that improve the decision‐making process and the outcomes of surgical procedures. In real time, AI can be used to aid in the analysis of huge volumes of data, the prediction of patient outcomes and the optimization of surgical methods [8].

Beside all euphoria for future robotic (r)evolution, we have to keep in mind that AI‐robotics first has to prove its benefit for clinical outcome and patient satisfaction in each and every patient. If some patients have poorer outcome than with conventional TKA, the journey of decision support will be stopped before it has really started. Therefore, solid data collection is of enormous importance. For example, it has to be controlled whether a specific alignment philosophy was performed according to the definitions or modified by the surgeon, otherwise the real impact of the different alignment strategies cannot be identified. Even this small aspect makes it obvious that it is of highest importance that interpretation of data collection will be performed by teams of medical experts and surgeons instead of medtech companies only.

In conclusion, TKA is on the verge of being revolutionized by AI, which will make it possible for robots to have a clinical influence in areas where navigation systems have been rendered ineffective. Robotic systems that are powered by AI have the potential to improve functional outcomes, boost precision and tailor procedures to the specific anatomy of each unique patient. Through the utilization of real‐time data and prediction algorithms, AI‐guided robots have the potential to overcome the limits of conventional approaches, thereby establishing a new benchmark for TKA. For us knee surgeons, it is of utmost importance to lead the process of AI tool integration in the OR, such as in robotic systems. Only then, we will still understand the rationale behind these algorithms, which is of utmost importance for clinical application and only then we can guide our patients in their clinical decisions. In future, every medtech company will have their own algorithmic AI‐driven pathway, which will impact the daily life of our patients.

## CONFLICT OF INTEREST STATEMENT

The authors declare no conflict of interest.

## ETHICS STATEMENT

The authors have nothing to report.
